# Therapeutic Alliance in Technology-Based Interventions for the Treatment of Depression: Systematic Review

**DOI:** 10.2196/17195

**Published:** 2020-06-11

**Authors:** Eileen Wehmann, Moritz Köhnen, Martin Härter, Sarah Liebherz

**Affiliations:** 1 Department of Medical Psychology Center for Psychosocial Medicine University Medical Center Hamburg-Eppendorf Hamburg Germany

**Keywords:** therapeutic alliance, depression, technology-based intervention, treatment

## Abstract

**Background:**

There is growing evidence that technology-based interventions (TBIs) are effective for the treatment of depression. As TBIs are gaining acceptance, a question arises whether good therapeutic alliance, considered a key aspect of psychotherapy, can be established without or with minimal face-to-face contact or rather changes if blended concepts are applied. While therapeutic alliance has been studied extensively in the context of face-to-face therapy, only few studies have reviewed evidence on alliance ratings in TBIs.

**Objective:**

The purpose of this study was to examine therapeutic alliance in technology-based psychological interventions for the treatment of depression.

**Methods:**

We searched Cochrane Central Register of Controlled Trials (CENTRAL), MEDLINE, PsycINFO, PSYNDEX, CINAHL, clinical trial registers, and sources of grey literature for randomized controlled trials on TBIs in the treatment of adults with unipolar depression. All publications were selected according to prespecified criteria. Data were extracted by two independent reviewers.

**Results:**

A total of eight out of 98 studies (9.5%) included in the review on TBIs for depression considered therapeutic alliance as part of their evaluation. The available data covered eight different treatment conditions, including four stand-alone treatments (face-to-face psychotherapy, email, telephone, and internet program) and four combined treatments (face-to-face psychotherapy plus a smartphone app and an internet program combined with face-to-face psychotherapy, treatment as usual, or email/telephone). On average, patients rated the alliance positively across all groups. Importantly, no relevant group differences regarding therapeutic alliance sum scores were found in any of the studies. Five studies investigated the relationship between patients’ alliance ratings and treatment outcome, revealing mixed results.

**Conclusions:**

Our results suggest that it is possible to establish a positive therapeutic alliance across a variety of different TBIs for depression, but this is based on a small number of studies. Future research needs to determine on what basis therapeutic alliance is formed in settings that do not allow for additional nonverbal cues, perhaps with adapted instruments to measure therapeutic alliance.

**Trial Registration:**

PROSPERO International Prospective Register of Systematic Reviews CRD42016050413; https://www.crd.york.ac.uk/prospero/display_record.php?ID=CRD42016050413)

**International Registered Report Identifier (IRRID):**

RR2-10.1136/bmjopen-2018-028042

## Introduction

### Background

Globally, over 300 million people (point prevalence in 2015) are estimated to have depression, accounting for 4.4% of the world population [1]. Depression is the greatest contributor for both disability and suicide [1] and is associated with greatly increased health care costs [2]. Treatment of depression with face-to-face psychotherapy has been shown to be effective over the last decades [3]. There is growing evidence that technology-based interventions (TBIs) are also effective for the treatment of depression, with great clinical improvement and recovery after treatment [4-9].

TBIs differ according to various aspects, including the type of technology (eg, computer and smartphone), therapeutic rationale (eg, cognitive behavior therapy and behavioral activation), the clinical phase of depression management (waiting period, acute treatment, and aftercare), and the amount of human support [10]. The degree of support as well as the delivery mode can range from a stand-alone self-administered treatment with no therapist contact at all to a blended treatment with active involvement of a therapist and regular face-to-face meetings.

Besides lowering the threshold to access treatment (eg, providing geographic and time-related flexibility) [11], TBIs, such as online therapy, provide an opportunity to reach people who refuse to seek traditional services, especially those who wish to remain anonymous [11,12].

Considering these aspects as well as the increasing interest in TBIs, a question arises whether adequate therapeutic alliance, considered a key aspect of psychotherapy, can be established without or with minimal face-to-face contact or rather changes when blended concepts are applied.

Therapeutic alliance can be viewed as the “quality of partnership and mutual collaboration between a therapist and client” [13], albeit a generally valid definition of the construct does not exist. According to Bordin [14], good therapeutic alliance is characterized by an agreement between the therapist and patient on the goals of the therapy, the tasks to be performed, and their emotional bond (eg, if the patient feels respected and appreciated by the therapist). Depending on the type of technology, the communication between the therapist and patient can be synchronous (telephone or video conference) or asynchronous (email or web-based program). Moreover, different technologies provide different cues about the therapist, such as visual (video conference) and auditory (telephone) cues, which may influence the emotional bond. This complexity needs to be incorporated when describing findings concerning therapeutic alliance, as pointed out in a recent review by Berger [15].

There are different instruments to assess therapeutic alliance, and the most frequently used instrument is the Working Alliance Inventory (WAI) [16,17], which includes the three aspects of *goal*, *task*, and *bond*. These instruments can be administered at different time points and are based on the patient’s, the therapist’s, or an observer’s point of view.

Positive alliance ratings are good predictors of treatment success in traditional face-to-face psychotherapy, even when controlling for other possible confounders, such as prior symptom change [13,18,19].

While therapeutic alliance has been studied extensively in the context of face-to-face therapy [13], only few studies have reviewed the evidence on alliance ratings in TBIs [17,20]. In a meta-analysis of 295 studies, Flückiger et al [17] recently showed that alliance ratings in internet-based programs are similar to those in face-to-face settings. They further revealed that the effect size of the relationship between alliance and treatment outcome is comparable in TBIs and face-to-face settings. The authors reported that most of the studies investigating alliance in internet-based interventions relied on the WAI. However, this review focused on internet-based interventions only and did not refer specifically to people diagnosed with depression. There are randomized controlled trials (RCTs) addressing therapeutic alliance in TBIs for people with depression [21,22]. However, there is no published systematic review on this topic.

### Aim

The purpose of this study was to examine therapeutic alliance in TBIs for the treatment of depression in a systematic review, considering the following research questions: (1) How many of the studies included in the review consider therapeutic alliance in their evaluation? (2) How is therapeutic alliance assessed? (3) How is the quality of therapeutic alliance rated across different interventions? (4) Is there a relationship between therapeutic alliance and treatment outcome?

## Methods

### Design

This study is part of a preregistered systematic review (PROSPERO registration number: CRD42016050413) on the comparative effectiveness of *T*echnology-based *I*nterventions in different steps of *De*pression *Ca*re (TIDECA). The corresponding protocol has been published [23] and includes more detailed descriptions. This review is in accordance with the standards of the Cochrane Collaboration [24] and is reported in line with the Preferred Reporting Items for Systematic Reviews and Meta-Analyses (PRISMA) statement [25].

### Search Strategy

We searched the following key databases: Cochrane Central Register of Controlled Trials (CENTRAL), MEDLINE, PsycINFO, PSYNDEX, and CINAHL. The search was not limited by date, language, or publication status. Supplementary material of the study protocol [23] includes applied search strategies of key databases. We further searched clinical trial registers (ClinicalTrials.gov, International Clinical Trial Registry Platform, and German Clinical Trial Register) and sources of grey literature (Open Grey, Trip Database, ProQuest Dissertations & Theses Abstract and Indexing, and specialized registers of Institute for Scientific Information Web of Science). Additionally, all first authors of the included publications were contacted for supplementary information on further published and unpublished trials and specific study information or the status of on-going studies, which were identified as published study protocols or preregistered trials.

### Inclusion and Exclusion Criteria

We applied PICOS categories (Population; Intervention; Comparator; Outcome; Study design) [24,26] to define the inclusion and exclusion criteria for our study. All abstracts were screened according to prespecified criteria. We included studies only if (1) they were cluster RCTs, (2) participants were 18 years or older, (3) participants had a diagnosis of unipolar depression based on a formal classification system, (4) mental or somatic comorbidities were not the main focus of the intervention or study, and (5) the active intervention included a TBI based on an explicit psychotherapeutic theory and aimed to improve depressive symptoms. For more details, refer to the study protocol [23].

### Selection Procedure

**Figure 1:**
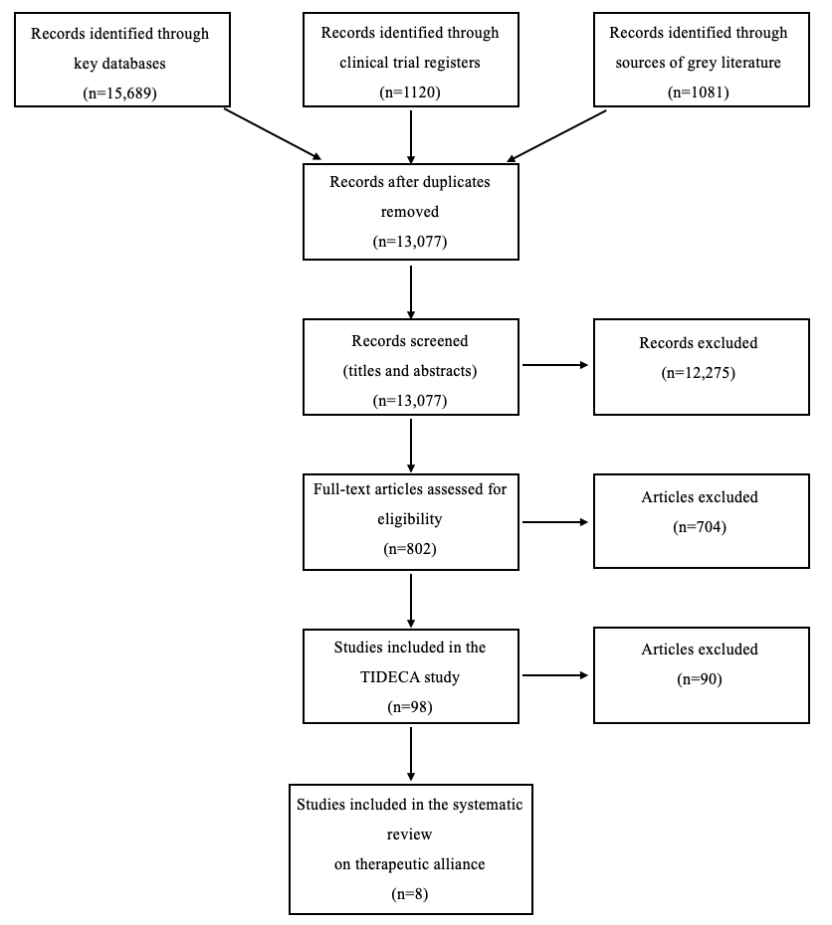
PRISMA flow diagram. TIDECA: Technology-based Interventions in different steps of Depression Care.

The selection process is presented in Figure 1. The search yielded at total of 13,077 records after duplicates were removed. Two authors (MK and SL) independently screened the first 100 records for inclusion. Since the interrater reliability for this sample was high (98%), one author (MK) screened the remaining records in the course of the title/abstract screening and the second author (SL) assessed publications labelled as “unclear.” Selected full-text publications (n=802) were subsequently screened for inclusion by two independent reviewers (MK and MD). Discrepancies were resolved by discussion with a third reviewer (SL). A total of 98 publications fulfilled all inclusion criteria for the TIDECA study and were finally screened to determine whether they included data on the *therapeutic working alliance relationship*. Eight publications were included in this review.

### Data Extraction

We developed a standardized data collection sheet, which was piloted on three studies. The sheet collected the following data: (1) general data (eg, year of publication), (2) methodological information (including risk of bias assessment), (3) demographic and clinical sample characteristics, (4) classification of the clinical phase of depression management (waiting period, acute treatment, and aftercare), (5) delivery mode (eg, blended treatment), (6) treatment characteristics, (7) sample size and study flow, (8) primary and secondary outcome data, and (9) data on therapeutic alliance, including measuring instrument, rating of therapeutic alliance for all included groups (means, standard deviations, and *P* values for available group differences), and data on the relationship between therapeutic alliance and treatment outcomes (*P* values). Data were extracted by two independent reviewers (EW and MK), and inconsistencies were resolved by discussion or by involving a third reviewer (SL).

### Data Analysis

Given the heterogeneity of the studies included, qualitative (respectively descriptive) synthesis of the evidence was performed, rather than a meta-analysis.

Descriptive statistics were used to present means, standard deviations, and percentage scores. Standard deviations were derived from standard errors, if not provided otherwise. We decided to focus on alliance ratings after the treatment had started (instead of pretreatment ratings) in order to detect possible group differences based on the different interventions. If several time points after the start of treatment were reported in the studies, we selected the earliest alliance ratings, in line with previous findings showing that therapeutic alliance is established at an early stage of therapy [27]. Given that only a small number of studies provided the therapists’ ratings of therapeutic alliance and all studies provided the patients’ ratings, the results focused on the patients’ ratings.

### Quality Appraisal

The risk of bias assessment was evaluated by two independent reviewers (EW and MK) for the included studies according to the Risk of Bias Tool of the Cochrane Handbook for Systematic Reviews [24] (including the domains random sequence generation, allocation concealment, blinding of participants and personnel, blinding of outcome assessment, incomplete outcome data, selective outcome reporting, and other bias). In line with a previous operationalization [28], we specified the domain “other bias” using the following three additional categories: insufficient treatment adherence, allegiance bias, and attention bias. Interrater reliability was calculated to be 77%. Discrepancies were resolved by discussions between the reviewers (EW and MK) or by involving a third reviewer (SL).

### Protocol Changes

Since the applied literature search was very comprehensive, we did not perform an additional systematic forward and backward reference search.

## Results

### Study Characteristics

Among the selected studies in the TIDECA review, 9.5% (8/98) considered therapeutic alliance as part of their evaluation. As displayed in Table 1, studies examining therapeutic alliance were published within the last decade and were mainly located in Europe, except for one study from the United States [29-36]. The sample size of these studies ranged from 38 to 325 participants. The technological delivery modes in the intervention groups included email, web-based programs, telephone, and a smartphone app. Waitlists, face-to-face psychotherapy, treatment as usual (TAU) combined with a waitlist, and an active control group with TAU were used as controls. Among the studies, four used a TBI as a stand-alone intervention, three implemented a blended treatment (ie, combining TBIs with face-to-face therapy), and one conducted enhanced stand-alone interventions (ie, combining TBIs with TAU without following a specific concept). The therapeutic rationale of applied TBIs was based on behavioral psychotherapy in all studies. The length of the treatment ranged from 8 to 18 weeks. The qualifications of the therapists varied from MSc students of clinical psychology and licensed psychotherapists to PhD-level psychologists.

Notable differences were observed with regard to the degree of therapist guidance. Therapists in predominantly self-help interventions focused on providing feedback, validation, reinforcement, and encouragement to continue with the program. In predominantly therapist-administered interventions, the therapy was delivered by therapists, whereas interventions based on self-help did not involve any therapists.

**Table 1:** Study characteristics.

Study, authors (publication year)	Study location	N^a^	Study arms	Delivery mode	Therapeutic rational of TBI^b^	Length (weeks)	Qualification of the therapists	Degree of guidance^c^
Andersson et al (2012) [29]	Sweden	88	Email support vs internet program vs waitlist	Stand-alone intervention	CBT^d^	8	MSc students of clinical psychology	Predominantly therapist administered (email) and predominantly self-help (internet)
Berger et al (2018) [30]	Germany	98	Internet program + F2F^e^ vs F2F	Blended treatment	CBT	12	Licensed psychotherapists	Predominantly therapist administered
Lindner et al (2014) [31]	Sweden	38	internet program + telephone support vs internet program + email support	Stand-alone intervention	BA^f^ + ACT^g^	8	MSc students of clinical psychology	Predominantly self-help
Ly et al (2015) [32]	Sweden	93	Smartphone + F2F vs F2F	Blended treatment	BA	9 (blended) and 10 (control)	MSc students of clinical psychology	Predominantly self-help
Meyer et al (2015) [33]	Germany	163	Internet program + TAU^h^ vs TAU + waitlist	Enhanced stand-alone intervention	CBT	13.05	No therapists involved	Self-administered
Steinmann et al (2019) [34]	Germany	59	Telephone + F2F + letters vs telephone + F2F	Stand-alone intervention	CBT	9-13	Licensed psychotherapists	Predominantly therapist administered
Stiles-Shields et al (2014) [35]	USA	325	Telephone vs F2F	Stand-alone intervention	CBT	18	PhD-level psychologists	Predominantly therapist administered
Zwerenz et al (2017) [36]	Germany	229	Internet program + TAU vs active control + TAU	Blended treatment	CBT	12	No therapists involved	Self-administered

^a^N: number of participants randomized.

^b^TBI: technology-based intervention.

^c^Based on the study by Newman et al [37].

^d^CBT: cognitive behavioral therapy.

^e^F2F: regular face-to-face psychotherapy.

^f^BA: behavioral activation.

^g^ACT: acceptance and commitment therapy.

^h^TAU: treatment as usual.

### Risk of Bias Assessment

**Figure 2:**
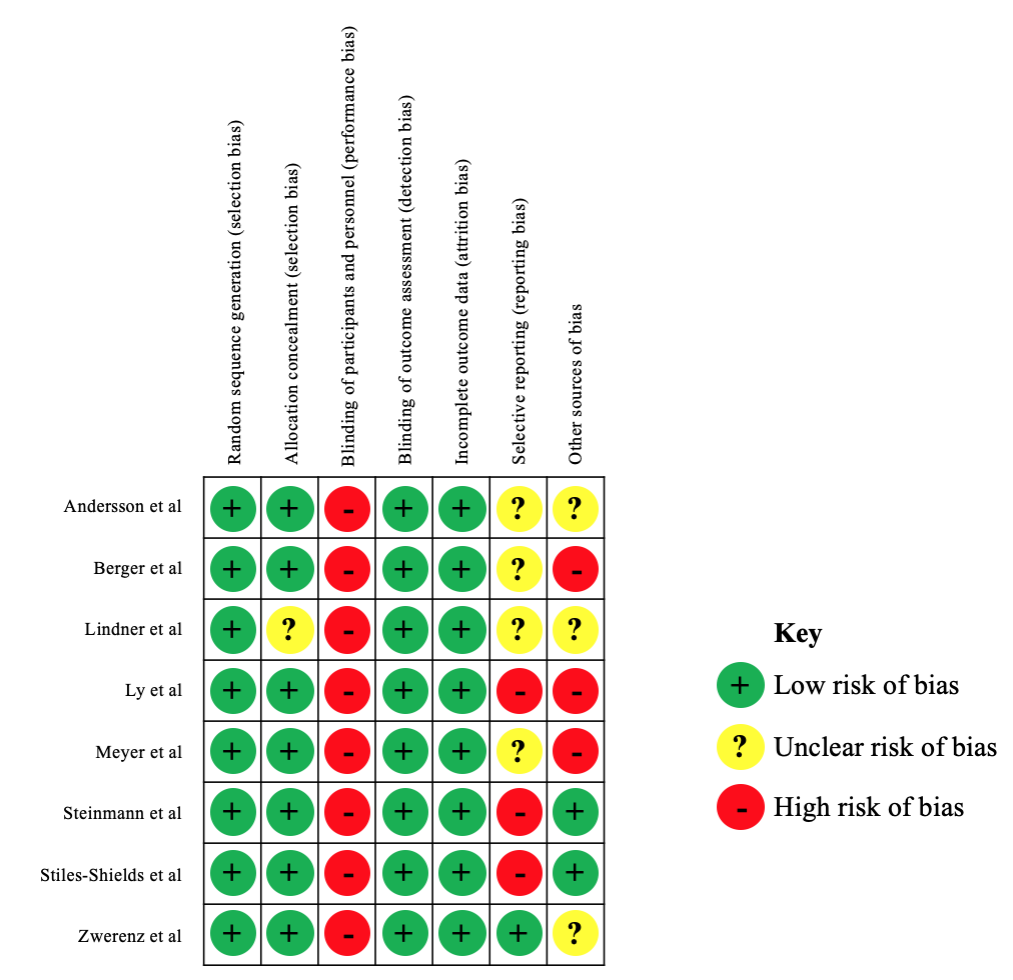
Risk of bias assessment. Other sources involved insufficient treatment adherence, allegiance bias or conflict of interest, and attention bias.

Overall, the risk of bias assessment showed a low risk of bias for selection bias, detection bias, and attrition bias (Figure 2). Given the nature of the studies, blinding of participants and personnel was not possible, thus creating a high risk for performance bias. Selective reporting (reporting bias) was unclear or high in all but one study, mainly due to unexplained or unjustified discrepancies between the protocol or the trial registration and the reported measures. Other sources of bias were unclear or high in all but two studies.

### Sample Characteristics

Table 2 provides an overview of the sociodemographic characteristics of the sample. The sample is rather typical for psychotherapeutic studies, with a mean participant age of 43.9 (SD 13.9) years and the majority of participants being female (769/1,093, 70.4%), being employed (554/941, 58.9%), and having a high level of education (672/1034, 65.0%). All studies included only participants who had a clinical diagnosis of depression, except for the study by Meyer et al [33], which had a sample with 84% (137/163) of patients having depression.

**Table 2:** Sample sociodemographic characteristics.

Characteristic		Value^a^ (N=1093^b^)	
Age (years)		43.9 (13.9)	
Sex (females)		769 (70.4%)	
**Occupation**			
	Employed (full and part time)		554 (58.9%)
	Unemployed (including retired and disabled)		303 (29.3%)
	Undergoing training (students and trainees)		41 (9.0%)
**Education**			
	Low educational level (<9 years)		42 (4.1%)
	Middle educational level (9-11 years)		297 (28.7%)
	High educational level (≥12 years)		672 (65.0%)
**Marital status**			
	Married/with partner		480 (46.4%)
	Single/without partner (including divorced and widowed)		329 (46.4%)

^a^Data are presented as mean (SD) or n (%).

^b^Values refer to the available data. Some studies did not report in line with our subcategories (missing data: occupation, four studies; education, one study; marital status, two studies).

### Assessment of Therapeutic Alliance

Table 3 summarizes how therapeutic alliance was assessed in the included studies. Five studies [29-32,35] relied on the WAI in its original format of 36 items (7-point Likert scale) [16], the short form version (WAI-S) with 12 items (7-point Likert scale) [38], or the short form-revised version (WAI-SR) with only positively worded items (5-point Likert scale) [39]. All five studies reported the total score, and three studies [29,30,35] further reported the subscores of the WAI (task, goal, and bond).

Two studies relied on the original Helping Alliance Questionnaire [40]; one reported the total score [36], whereas the other reported the subscore for collaboration or bond [34]. One study [33] assessed therapeutic alliance using the 11-item Helping Alliance Questionnaire (HAQ-11) [41], with a 6-point response score. The mean total score of the HAQ-11 was converted to a percentage score that indicated how many participants viewed the alliance as positive.

All studies used the client-rated version of the assessment tools, and three studies [30,35,36] also utilized therapist ratings. While all studies scheduled an early or mid-treatment assessment of the alliance, ranging from 2 to 6 weeks of treatment, some studies included pretreatment and follow-up evaluations as well. Notably, Steinmann et al [34] reported alliance ratings at study intake only, and Zwerenz et al [36] administered the HAQ to measure therapeutic alliance in the TAU arm but not in the TBI arm. Therefore, these data were not considered in the subsequent results.

**Table 3:** Assessment of therapeutic alliance.

Study, authors (publication year)	Assessment tool	Measures reported	Rater	Time of assessment	Total treatment length
Andersson et al (2012) [29]	WAI^a^	Total score + 3 subscores (task, goal, and bond)	Client	After 3 weeks	8 weeks
Berger et al (2018) [30]	WAI-Short Revised	Total score + 3 subscores (task, goal, and bond)	Client + therapist	After 6/12 weeks	12 weeks
Lindner et al (2014) [31]	WAI-Short Form	Total score	Client	After 2/8/12 weeks	8 weeks
Ly et al (2015) [32]	WAI-Short Form	Total score	Client	Pre-treatment/after 3 weeks	9 weeks (10 weeks control)
Meyer et al (2015) [33]	HAQ-11^b^	Total score	Client	After 3 weeks	13.05 weeks^c^
Steinmann et al (2019) [34]	HAQ^d^	Subscore (collaboration or bonding)	Client	Study intake	9-13 weeks
Stiles-Shields et al (2014) [35]	WAI-Short Revised	Total score + 3 subscores (task, goal, and bond)	Client + therapist	At 4/14 weeks	18 weeks
Zwerenz et al (2017) [36]	HAQ	Total score	Client + therapist	Study intake/end of TAU^e^	12 weeks

^a^WAI: Working Alliance Inventory.

^b^HAQ-11: 11-item Helping Alliance Questionnaire.

^c^A period of 3 months was converted to weeks with a factor of 4.35 weeks per month for comparability.

^d^HAQ: Helping Alliance Questionnaire.

^e^TAU: treatment as usual.

### Ratings of Therapeutic Alliance

The patients’ ratings of therapeutic alliance (between week 2 and 6) are displayed in Table 4. The available data cover eight different treatment conditions, including four *stand-alone treatments* (face-to-face psychotherapy, email, telephone, and internet program) and four *combined treatments* (face-to-face psychotherapy plus a smartphone app and an internet program combined with face-to-face psychotherapy, treatment as usual, or email/telephone).

On average, patients rated the alliance positively across all groups. Importantly, no relevant group differences regarding therapeutic alliance sum scores were found in any of the studies. Andersson et al [29] found a significant difference (*P*=.04) between email-based therapy and the use of a self-help internet program when comparing the subscore *goal*, with higher scores in the email condition. However, no relevant group differences regarding the other subscores where found.

Berger et al [30] and Stiles-Shields et al [35] further analyzed therapists’ alliance ratings and found no statistically significant group differences between face-to-face psychotherapy and face-to-face psychotherapy combined with an internet program (*P*>.09) or between face-to-face psychotherapy and telephone treatment (*P*>.05).

**Table 4:** Patients’ ratings for therapeutic alliance.

Study, authors (publication year) and subscale		Alliance ratings^a^, mean (SD)								Group differences^d^	
		F2F^b^	F2F + smartphone	Internet program	Internet program + F2F	Internet program + TAU^c^	Email	Internet program + email/phone	Telephone		
**Andersson et al (2012)** [29]											
	Total	N/A^e^	N/A	5.25 (0.82)	N/A	N/A	5.58 (0.82)	N/A	N/A	>.05	
	Task	N/A	N/A	5.19 (0.84)	N/A	N/A	5.23 (0.83)	N/A	N/A	>.05	
	Bond	N/A	N/A	5.47 (0.97)	N/A	N/A	5.86 (0.91)	N/A	N/A	>.05	
	Goal	N/A	N/A	5.08 (0.92)	N/A	N/A	5.63 (0.86)	N/A	N/A	.04^f^	
**Berger et al (2018)** [30]											
	Total	3.48 (0.88)	N/A	N/A	3.64 (0.59)	N/A	N/A	N/A	N/A	>.03^g^	
	Task	3.27 (0.89)	N/A	N/A	3.42 (0.63)	N/A	N/A	N/A	N/A	>.03^g^	
	Bond	3.60 (1.00)	N/A	N/A	3.98 (0.64)	N/A	N/A	N/A	N/A	>.03^g^	
	Goal	3.55 (0.99)	N/A	N/A	3.52 (0.74)	N/A	N/A	N/A	N/A	>.03^g^	
**Lindner et al (2014)** [31]											
	Total	N/A	N/A	N/A	N/A	N/A	N/A	58.37 (10.55)	N/A	.6	
**Ly et al (2015)** [32]											
	Total	65.7 (11.3)	63.5 (9.6)	N/A	N/A	N/A	N/A	N/A	N/A	.75-.37	
**Meyer et al (2015)** [33]									N/A		
	Total	N/A	N/A	N/A	N/A	71.0%	N/A	N/A	N/A	N/A	
**Stiles-Shields et al (2014)** [35]											
	Total	49.9 (7.57)	N/A	N/A	N/A	N/A	N/A	N/A	49.7 (7.45)	.78	
	Task	23.3 (4.26)	N/A	N/A	N/A	N/A	N/A	N/A	23.4 (4.15)	.86	
	Bond	21.9 (5.21)	N/A	N/A	N/A	N/A	N/A	N/A	22.0 (5.13)	.76	
	Goal	16.5 (2.48)	N/A	N/A	N/A	N/A	N/A	N/A	15.9 (2.44)	.053	

^a^Means and standard deviations are displayed, except for the study by Meyer et al, where the percentage of participants rating the alliance as positive is displayed.

^b^F2F: regular face-to-face psychotherapy.

^c^TAU: treatment as usual.

^d^*P* values based on *t* tests (Andersson et al and Ly et al), Mann-Whitney *U* tests (Berger et al), repeated measures analysis of variance (Lindner et al), and least square estimated means (Stiles-Shields et al).

^e^N/A: not applicable.

^f^Statistically significant.

^g^Not statistically significant after Bonferroni correction.

### Relationship Between Therapeutic Alliance and Treatment Outcome

Five studies [29,30,32,33,35] investigated the relationship between patients’ alliance ratings and treatment outcome (Table 5).

Berger et al [30] found a statistically significant positive association between patients’ alliance ratings at 6 weeks and treatment outcome in the face-to-face psychotherapy group (*P*<.05) but not in the combined treatment group. They further showed that residual gain scores of depression were predicted by the patients’ alliance rating at 12 weeks in both the regular face-to-face therapy group (*P*<.05) and combined group (*P*<.01). The therapists’ ratings at 6 weeks showed no significant association with treatment outcome for either the regular (*P*=.61) or combined treatment (*P*=.08). The therapists’ ratings at 12 weeks were significantly associated with treatment outcome in the combined group (*P*<.05) but not in the regular face-to-face therapy group (*P*=.60).

Ly et al [32] reported a significant positive association between patients’ working alliance scores and changes in treatment outcome for the blended treatment (*P*<.05) but not for the regular face-to-face psychotherapy.

Meyer et al [33] found a positive correlation between the patients’ HAQ-11 scores after 3 weeks of treatment with the internet program plus treatment as usual and symptom reduction. This correlation remained significant (*P*<.02) when controlling for early symptom change.

Anderson et al [29] and Stiles-Shields et al [35] found no relevant relationship between patients’ alliance ratings and treatment outcome.

**Table 5:** Relationship between patients’ therapeutic alliance ratings and treatment outcome.

Study, authors (publication year)	F2F^a^	F2F + smartphone	Internet program	Internet program + F2F	Internet program + TAU^b^	Email	Internet program + email/phone	Telephone
Andersson et al (2012) [29]	N/A^c^	N/A	0^d^	N/A	N/A	N/A	N/A	N/A
Berger et al (2018) [30]	+^e^	N/A	N/A	0	N/A	N/A	N/A	N/A
Ly et al (2015) [32]	0	+	N/A	N/A	N/A	N/A	N/A	N/A
Meyer et al (2015) [33]	N/A	N/A	N/A	N/A	+	N/A	N/A	N/A
Stiles-Shields et al (2014) [35]	0	N/A	N/A	N/A	N/A	N/A	N/A	0

^a^F2F: regular face-to-face psychotherapy.

^b^TAU: treatment as usual.

^c^N/A: not applicable.

^d^No significant relationship between therapeutic alliance and treatment outcome.

^e^Statistically significant positive relationship between therapeutic alliance and treatment outcome. Significance based on Spearman correlation (Berger et al, *P*<.01) and mixed effects models (Ly et al, *P*=.00-.05; Meyer et al, *P*<.01).

## Discussion

### Principal Findings

This study investigated if and how therapeutic alliance ratings were considered in RCTs on different technology-based psychological interventions for depression. Out of the 98 studies included in the TIDECA review, 8 (9.5%) investigated therapeutic alliance. A previous review on e-therapy for different mental health diagnoses found that 1.3% of the included studies considered measures of therapeutic alliance [20]. Notably, the studies included in that review were published between 2002 and 2010, whereas the studies in our review were published between 2012 and 2019. This result may suggest an increased interest in the subject matter; however, it is difficult to directly compare the numbers, since the inclusion criteria of both studies were not identical. Our results showed that the assessment of therapeutic alliance was based on either the WAI or HAQ and that all studies used the patients’ ratings, whereas only three studies reported the therapists’ ratings as well. Furthermore, this study analyzed therapeutic alliance ratings across different interventions considering factors (eg, the degree of therapeutic guidance) that may affect therapeutic alliance, as well as the relationship between therapeutic alliance and treatment outcome.

It was shown that in a face-to-face psychotherapy setting, the therapeutic relationship was not rated differently when adding an internet-based program as an adjunctive treatment tool. Further, a setting with reduced (four instead of 10) face-to-face sessions and a supportive smartphone app also showed no relevant differences compared with a control setting (10 face-to-face sessions and no smartphone app) regarding therapeutic alliance. Replacing all face-to-face sessions with telephone therapy revealed no relevant difference in alliance ratings. This is especially notable as telephone communication eliminates all visual cues.

In guided internet-based programs, no difference regarding therapeutic alliance was found between guidance via email and guidance via telephone. Thus, in this specific setting, vocal cues and synchronous communication, as provided by the telephone, did not result in other therapeutic alliance ratings compared with asynchronous communication and missing vocal cues. Further, guided internet programs showed alliance ratings similar to those for individualized email therapy.

Overall, therapeutic alliance was rated positively, regardless of the type of technology applied. Notably, this also applied to a setting with no therapist contact at all [33], although it is unclear what exactly is reflected by alliance measures between a person and a program.

Our results are based on one study for each treatment comparison; thus, conclusions need to be considered with caution. Further, therapeutic alliance was assessed at different time points, ranging from 2 to 6 weeks after baseline, which adds heterogeneity to the sample. Thus, different alliance ratings may relate to how the relationship was perceived at a specific time point during the treatment, rather than reflecting differences between the interventions themselves. With this in mind, our results suggest that in the context of TBIs for depression, visual and vocal cues, synchronous communication, and physical presence of the therapist are not requirements for developing good therapeutic alliance. This finding is especially interesting in light of the idea that nonverbal behavior is a key factor of relationship formation between patients and therapists [42]. Possibly, there are other factors that compensate for the lack of nonverbal cues, such as more flexibility when accessing therapeutic modules in web-based programs or smartphone apps. Additionally, there are limited studies about the influence of nonverbal cues on the therapeutic relationship [43]; thus, the relevance of these cues for building an adequate alliance is unclear.

We found mixed results concerning the relationship between therapeutic alliance and treatment outcome (eg, alliance ratings in face-to-face therapy were closely related to the treatment outcome in one study but not in another). It has been argued that alliance ratings are not directly related to treatment outcome, but rather represent a third variable, such as early improvement and treatment motivation [44]. This notion was not confirmed by the results of the study by Meyer et al [33], which showed that early alliance ratings predicted treatment outcome, even when controlling for early symptom change.

### Strengths and Limitations

Our review was conducted in line with the Cochrane guidelines, and studies were selected according to prespecified criteria, which were previously published in the study protocol, reflecting high methodological standards. The strict application of inclusion and exclusion criteria reduced the overall number of studies considered in this review. All studies were conducted in Western countries, mainly Europe (Sweden and Germany), and one study was conducted in the United States. These countries share similar communication patterns (eg, relying highly on semantic meaning rather than contextual and nonverbal cues) [45]. Thus, it is unclear whether our results can be extended to non-Western countries, especially in the context of TBIs, where visual and vocal cues are eliminated (eg, email and web-based programs).

There are some additional considerations when interpreting our results. First, the majority of the studies relied upon the WAI for the assessment of therapeutic alliance. As recently pointed out [46], this instrument has not been developed and tested for use in technology-based interventions and may need some adaptions to identify setting-specific influences. This may apply to the specific wording of the instrument (eg, *program* instead of *therapist*), as well as the content of the questions (eg, the goals of the treatment are usually not discussed in a self-help program). Thus, it is possible that future studies utilizing a tool that is specifically developed and validated in this context may show divergent results.

Second, not all studies specifically reported whether any additional contact between therapists and patients occurred outside of the treatment setting. For example, if a patient is assigned to an email therapy group but meets the therapist during the recruitment or initial assessment process, additional exposure to visual and vocal cues could influence the formation of a therapeutic bond. Thus, we encourage future studies to take this point into consideration by reporting all contacts between therapists and patients.

Third, most patients were recruited via advertisements. Previous research has shown that patients recruited from nonclinical settings have a more positive attitude toward internet interventions for the treatment of depression than patients recruited from clinical settings [47]. Further, therapists that agreed to participate in the studies may have been subject to a similar selection bias. Thus, the results may not be transferrable to clinical practice, where more skeptical individuals (concerning TBIs) are present.

Fourth, only three out of eight studies took the therapists’ perception of therapeutic alliance into consideration. Previous studies have shown that therapists and patients may judge the alliance differently [13]; thus, it would be favorable to include both measures in future studies.

### Implications for Clinical Practice and Future Research

As the field may continue to expand and more therapists may consider technology-based treatment options, it is important to further investigate on what basis the alliance is formed in settings that do not allow for additional cues, such as facial expression and tone. For example, in an RCT with several arms, each eliminating a different component of face-to-face communication (eg, visual or auditory cues), researchers could investigate if and how therapeutic alliance is affected. Importantly, these studies should include both the therapists’ and patients’ perspectives. Further, it will be important to replicate the finding that early alliance ratings may predict treatment outcome independently from early symptom change. Such studies will require more frequent measurements of therapeutic alliance, symptom change, and alternative factors that could relate to treatment outcome, such as pretreatment motivation, in order to establish the relation of these aspects. Moreover, research needs to address how personal preferences and attitudes toward TBIs may interact with the formation of a therapeutic alliance, both from the patient’s and therapist’s perspective. Finally, it remains unclear what exactly is measured in settings without any therapist contact at all. Possibly, the alliance between a person and a technology-based program reflects less of the emotional aspect of therapeutic alliance and is more related to how the program matches an individual’s goals and expectations of the tasks required.

### Conclusion

This review shows that studies on therapeutic alliance in TBIs for the treatment of depression are still limited, especially regarding therapists’ alliance ratings. Taking into account different degrees of therapeutic guidance, qualifications of therapists, modes of delivery, and types of technologies, the results of this review suggest that a positive therapeutic alliance can be established in TBIs for people with depression.

## Abbreviations

HAQ: Helping Alliance Questionnaire
HAQ-11: 11-item Helping Alliance Questionnaire
RCT: randomized controlled trial
TAU: treatment as usual
TBI: technology-based intervention
TIDECA: Technology-based Interventions in different steps of Depression Care
WAI: Working Alliance Inventory
WAI-S: Working Alliance Inventory short form version
WAI-SR: Working Alliance Inventory short form-revised version
